# “It’s a complex mesh”- how large-scale health system reorganisation affected the delivery of the immunisation programme in England: a qualitative study

**DOI:** 10.1186/s12913-016-1711-0

**Published:** 2016-09-15

**Authors:** Tracey Chantler, Saumu Lwembe, Vanessa Saliba, Thara Raj, Nicholas Mays, Mary Ramsay, Sandra Mounier-Jack

**Affiliations:** 1 London School of Hygiene & Tropical Medicine, Faculty of Public Health & Policy, London, UK; 2 Immunisation, Hepatitis & Blood Safety Department, National Infection Service, Public Health England, 61 Colindale Avenue, London, UK; 3 Bristol City Council, Public Health, Bristol City Council, City Hall (formerly The Council House), College Green, Bristol, BS1 5TR UK

**Keywords:** Delivery of health services, Health reforms, Immunisation, Organisational change, Public health, Qualitative research

## Abstract

**Background:**

The English health system experienced a large-scale reorganisation in April 2013. A national tri-partite delivery framework involving the Department of Health, NHS England and Public Health England was agreed and a new local operational model applied. Evidence about how health system re-organisations affect constituent public health programmes is sparse and focused on low and middle income countries. We conducted an in-depth analysis of how the English immunisation programme adapted to the April 2013 health system reorganisation, and what facilitated or hindered the delivery of immunisation services in this context.

**Methods:**

A qualitative case study methodology involving interviews and observations at national and local level was applied. Three sites were selected to represent different localities, varying levels of immunisation coverage and a range of changes in governance. Study participants included 19 national decision-makers and 56 local implementers. Two rounds of interviews and observations (immunisation board/committee meetings) occurred between December 2014 and June 2015, and September and December 2015. Interviews were audio recorded and transcribed verbatim and written accounts of observed events compiled. Data was imported into NVIVO 10 and analysed thematically.

**Results:**

The new immunisation programme in the new health system was described as fragmented, and significant effort was expended to regroup. National tripartite arrangements required joint working and accountability; a shift from the simpler hierarchical pre-reform structure, typical of many public health programmes. New local inter-organisational arrangements resulted in ambiguity about organisational responsibilities and hindered data-sharing. Whilst making immunisation managers responsible for larger areas supported equitable resource distribution and strengthened service commissioning, it also reduced their ability to apply clinical expertise, support and evaluate immunisation providers’ performance. Partnership working helped staff adapt, but the complexity of the health system hindered the development of consistent approaches for training and service evaluation.

**Conclusion:**

The April 2013 health system reorganisation in England resulted in significant fragmentation in the way the immunisation programme was delivered. Some of this was a temporary by-product of organisational change, other more persistent challenges were intrinsic to the complex architecture of the new health system. Partnership working helped immunisation leaders and implementers reconnect and now the challenge is to assess how inter-agency collaboration can be strengthened.

**Electronic supplementary material:**

The online version of this article (doi:10.1186/s12913-016-1711-0) contains supplementary material, which is available to authorized users.

## Background

### Introduction

Immunisation averts an estimated 2–3 million deaths globally every year but despite this achievement an estimated 21.8 million infants still miss out on essential vaccines, and coverage gaps persist between and within countries [1]. Increasing coverage is not just a question of addressing unmet need and increasing access to services, other factors can also affect the performance of immunisation programmes. Evidence from low and middle income countries suggests that effective leadership and regular training of health care staff can strengthen programmes [2, 3], whereas health reforms can lead to inequities in coverage and operational weaknesses [4, 5]. WHO’s ‘Global Routine Immunisation Strategies and Practices’ report stresses the importance of maintaining cohesive immunisation programmes that are well aligned with broader health systems [6]. This suggests a symbiotic relationship between the overarching health system and integral public health programmes, but what happens when the health system is reformed, how do these programmes adapt? In this study we sought to determine how a large-scale re-organisation of the English health and social care system (April 2013) affected a well performing, vertically oriented public health programme with a clear chain of command and implementation structures.

### The situation pre April 2013

Prior to April 2013 the English immunisation programme was amongst the best performing in high income countries [7]. In 2012–13 94.7 % of infants completed their primary course of DTaP/IPV/Hib and MMR coverage at age 2 was 91.2 % [8]. This MMR coverage was the highest achieved since the introduction of the vaccine in 1988, indicating that public confidence had been restored following the Wakefield controversy [9].

It is important to state that the health and social care system has undergone numerous reforms over the past decades [10]. In 1990 the National Health Service (NHS) and Community Care Act [11] resulted in the introduction of a purchaser-provider split. This reform made it easier for private providers to enter a competitive market for the provision of NHS services including immunisations. The concept of purchasing became known as commissioning and a somewhat artificial divide between purchasers/commissioners and providers was created.

### The April 2013 health system reorganisation in England

The NHS in England was reorganised following the enactment of the 2012 Health and Social Care Act (HSCA) on 1st April 2013 [12]. The stated objectives of this large-scale health and social care system reform were to empower patients, put primary care clinicians at the centre of service commissioning, free up providers to innovate, and provide a new focus on public health. The underlying rationale was to *“liberate the NHS from Ministerial control*” and *“free staff from excessive bureaucracy and top-down control”* [13]. As a result, significant changes were made to the structure and organisation of the health system. Responsibility for running the NHS was transferred from the Department of Health (DH) to NHS England, a new arms-length body. Primary Care Trusts (PCTs), which were accountable to DH and commissioned the majority of services for local geographical areas were abolished and replaced with general practitioner led Clinical Commissioning Groups (CCGs). Public Health England was established as a new executive agency of the DH, that incorporated the core health protection functions of the former Health Protection Agency, and brought together more than 70 organisations into a single public health service. However, some specific powers were delegated to local authorities (government bodies responsible for specific geographic areas), these are to give information and advice on appropriate health protection arrangements within their local area, and to provide a public health advice service to CCGs [14]. The changes took place against a backdrop of large cuts to NHS management costs resulting in new agencies performing their functions with restricted human and financial resources.

### Repercussions for the national immunisation programme

This reorganisation and in particular the delegation of functions to existing, modified and new organisations, had a knock-on effect on the distribution of responsibilities for immunisation. This represented a significant change from the previous arrangements whereby the PCT had been the sole organisation responsible for the commissioning, coordination and evaluation of immunisation. The responsibilities of former PCT staff (e.g. immunisation coordinators, immunisation programme managers), who had played a core role in supporting and performance managing the programme at the local level were distributed among various agencies, often covering much bigger geographies (Table 1). Local authorities were also required to work with PHE and local partners to ensure that threats to health, including vaccine preventable disease outbreaks, are understood and addressed and that the right preventative strategies are in place to tackle threats to the health of their population. This included providing assurance for the immunisation programmes that were now commissioned by NHS England [15, 16].

**Table 1:** Immunisation landscape post NHS reform 2013

Key system component	Responsible organisation – pre reforms	Responsible organisation – post reforms (April 2013)
Policy development, advice to ministers	Department of Health (national)	Department of Health (national)
Vaccine Procurement	Department of Health (national)	Public Health England (national)
Commissioning	Primary Care Trust (local)	NHS England (national)
• 16 national programmes • School based programmes		Local authorities (local) or NHS England (national)
Disease surveillance/Outbreak response	Health Protection Agency (national)	Public Health England (national) and NHS England (national)
Advocacy, communication and health promotion	Primary Care Trust (local)	Public Health England (national)
		Local authorities (local)
System coordination	Primary Care Trust (local)	NHS England (national)
Vaccine, Cold Chain and Logistics Management	Primary Care Trust (local)	Public Health England (national)
Vaccine Delivery	General Practitioners (local), NHS Community Trusts (local), other providers (local or national)	General Practitioners (local), NHS Community Trusts (local), other providers (local or national)
Child Health Information System (CHIS) and Data management	Primary Care Trusts through Child Health Information Systems (local)	Child Health Departments through CHISs (local)
Workforce training	Primary Care Trusts (local)	Health Education England (national)
Others: Needs assessments, scrutiny and system assurance.	Primary Care Trusts (local)	Local Authorities (local)
Others: Quality improvement (Duty of)	Primary Care Trusts (local)	Clinical Commissioning Groups (CCGs) (local but need to give assurance to NHS England which is national)

*Source*: Adapted from the immunisation and screening national delivery framework & local operating model (NHS E and PHE, 2013)

### The national delivery framework

As a result of the changes, the immunisation programme is now managed through a tripartite (three organisations: DH, NHS England and PHE) national delivery framework and a local operating model [17]. The national framework assigns DH responsibility for providing national strategic oversight, NHS England responsibility for commissioning services and PHE responsibility for providing scientific support. Commissioning intentions and budget requirements for the delivery of the immunisation programme are agreed annually by DH and NHS England, and published in a public health functions agreement referred to as Section 7a [18]. This legal agreement has to be approved by the NHS England Board and Secretary of State. PHE supports DH and NHS England in system leadership and planning, and has specific responsibilities for the implementation of the immunisation programme, the provision of service specifications for individual vaccine programmes (for details of these see pages 16–17 of [18]), the procurement of vaccines and immunoglobulins, and the provision of specialist advice and information at national and local level.

### The local operating model

At local level, PHE employs screening and immunisation teams (SITs) embedded within NHS England Local Teams covering different geographic areas. This means that SITs are accountable to both PHE and NHS England. SITs are responsible for providing local leadership, encouraging multi-agency working, ensuring high quality delivery of programmes based on national specifications, supporting commissioning, providing advice to the public and health professionals, and monitoring the performance of community and primary care providers [17]. CCGs are expected to support SITs particularly with quality improvement in primary care. Local government is responsible for providing independent scrutiny of the local immunisation programme delivery, ensuring it is responsive to local population needs, and commissioning community health services, such as school nursing and sexual health, which can include immunisation activities [16]. Specialist public health teams headed by a Director of Public Health were established within local government offices, commonly referred to as local authorities (LAs) and positions mainly filled by previous PCT public health staff [16].

In England, the majority of vaccines that form part of the routine schedule (see [19]), are given in primary care by practice nurses working in General Practitioner (GP) led surgeries. In the new health system, the GP contract which includes immunisation activities, is updated yearly by national NHS England primary care commissioners, whereas previously local PCTs had some input into these negotiations. The commissioning of community immunisation services (e.g. school nurses or designated immunisations teams delivering immunisations in schools) on the other hand can involve both NHS England Local Teams and LA Public Health Teams.

The system changes happened in the context of significant programmatic changes and increased workload for immunisers with the addition of rotavirus vaccine (2013) and meningococcal group B vaccine (2015) to the routine childhood immunisation programme; the introduction of the shingles vaccine for older adults (2013) and the phased extension of the influenza programme to children (2013).

In summary the health system reorganisation resulted in a reallocation of responsibilities for commissioning, delivering and assuring the national immunisation programme. The creation of new and revised agencies at a time of financial constraint and the re-deployment of the public health workforce in conjunction with significant changes to the immunisation schedule had significant managerial, operational and public health implications.

### The effects of large-scale organisational change on health systems

Implementing large-scale organisational change to health systems, commonly referred to as ‘big bang’ changes [20], is complex, difficult to manage, liable to generate unintended consequences [21, 22]. Specific negative effects of these types of changes include a loss of focus on services, delays in service improvement, difficulties in sharing good practice, loss of familiarity between different tiers of staff, reduced motivation levels and associated cynicism [23, 24]. Mitigating strategies include adopting a participatory and open style of leadership, building on familiar and valued ideas and activities, being aware of potential clashes in working cultures, and monitoring the impacts of the changes on the individuals and organisations [23, 25, 26]. The literature on how wider health system re-organisations affect constituent public health programmes, such as immunisation, is sparse and focused on low and middle income countries. It suggests that health sector reforms and related repercussions on immunisation strategies can lead to inequities in coverage [4], the loss of institutional memory and operational weaknesses [5].

### Purpose and framing of this study

The purpose of this study was to generate evidence about the effects of large-scale health system re-organisation on the delivery of a public health programme in a high-income country, and to document how these changes were managed and mitigated, particularly as immunisation leaders had voiced concerns about the potential associated risks of re-organisation [27, 28].

Our aim was to conduct an in-depth analysis of how the national immunisation programme had adapted to the April 2013 health system reorganisation and what facilitated or hindered the delivery of immunisation services in this context. As such, our motivation was to map the response of the immunisation programme to a ‘big bang’ (large-scale health care reform that went live on a single date) in order to identify lessons for ongoing and future change management in public health programmes. Specific objectives were to determine how the new national delivery framework and local operating model were being implemented in practice, how organisations and staff were managing change and adjusting to new roles and responsibilities, how the health system re-organisation had affected their capacity to deliver and manage the performance of the immunisation programme, and what mechanisms were being put in place to facilitate collaboration across organisations involved in the programme.

## Methods

### Design

Studies that seek to investigate the management and effects of change, need to use methods which allow for the process to be explored and understood [21]. Hence we chose to use a qualitative case study methodology [29, 30] involving interviews and observations of practice at national level and in three local implementation sites. The interviews with a wide range of participants from the different organisations involved in the delivery of the immunisation programme generated narratives of the process of organisational change allowing it to be understood [31, 32]. A similar approach has been used to analyse previous NHS reforms [33]. To complement these two methods, we also drew on key policy documents relating to the health system reforms and the new arrangements for delivery of the immunisation programme. Our interviewees were national representatives from tri-partite organisations responsible for programme oversight and local level programme implementers from three local sites. We chose to document the effects of the health reforms on the delivery of the immunisation programme at the macro and the meso levels (middle level organisations involved in delivering the immunisation programme to individual populations) in order to be able to examine how the new national delivery framework and local operating models were being applied at these different levels, and to document the vertical and horizontal relationships between and across these levels. At national level, PHE colleagues supported the identification of potential participants from PHE, DH, NHS England, professional bodies and the Joint Committee on Vaccination and Immunisation, who were involved in policy making, and providing leadership and strategic oversight for the immunisation programme. At local level, Screening and Immunisation Leads helped the research team map the implementation of the immunisation programme at the three sites in order to identify potential participants from SITs, NHS England, PHE Health Protection Teams, LA Public Health teams, CCGs and service providers (e.g. practice nurses). The local level implementation sites were selected to represent different geographical areas, varying levels of immunisation coverage and a range of changes in governance (see Table 2 for more details on these differences).

**Table 2:** Study Participants (*n* = 75)

National level decision-makers									
National Key Informants	Public Health England			NHS England	Department of Health	Professional Organisations (RCN^a^, ADPH^b^)	JCVI	Other	
	11			2	3	2	1	0	19
Sub totals									19
Local implementers									
	Profile	NHSE/PHE^c^	NHS England	Local Authority Public Health Teams	CCG^d^ members	Service providers^e^		Other	
Site 1	Rural, semi-urban and urban area, public health structures experienced major and minor changes, patches of high and low immunisation coverage	5	1	8	2	3		0	19
Site 2	Urban area, public health structures experienced moderate and minor changes, patches of medium and low immunisation coverage	8	3	3	1	10^f^		1 (Imms Trainer)	26
Site 3	Urban area, public health structures experienced moderate and minor changes, patches of medium and high immunisation coverage.	5	0	1	3 (also providers)	2 (1 works part time with LA Public Health Team)		0	11
Sub Totals		18	4	12	6	15		1	56
Totals									75

^a^Royal College of Nursing ^b^Association of Directors of Public Health^c^Screening and Immunisation Team members & PHE Health Protection Unit Leads^d^Clinical Commissioning Groups, ^e^Practice Nurses and Community Provider Immunisation Leads ^f^ Including one FGD with 9 Practice Nurses

### Data collection

Data was collected by a team of three researchers who approached potential participants by email and obtained their written informed consent prior to interviewing them. A first round of interviews and observations occurred between December 2014 and June 2015, and a second round between September and December 2015. The second round included interviews with new participants, identified iteratively as a result of previous interviews and observations, and some follow-up interviews and feedback discussions with existing participants to clarify emerging questions and find out about ongoing developments at national and local level. The complete data set comprised of observations of 3 national immunisation board meetings and 3 local immunisation board/committee meetings, and interviews (individual, peer and focus group) with 19 national level decision-makers and 56 local implementers (Table 2). The aim of the interviews was to gain insights into participants’ perspectives and experiences on pre-defined subject areas (Table 3, and see also Additional files 1, 2 and 3), whilst remaining flexible enough to encourage the exchange of other relevant content. Hence, additional topics were added as new questions emerged during data collection and analysis.

**Table 3:** Interview subject areas

Current roles and responsibilities for immunisation in the new health system;	
Changes in the way the immunisation programme is managed and delivered post April 2013;	
Management of change (consultation, information, coordination), and the effects of the changes;	
Adaptation of the immunisation programme, current functioning and performance against local and national immunisation standards;	
Opportunities, strengths and weaknesses of the new system, nationally and locally.	

The recruitment of providers was challenging since the nature of their work meant they were less able to engage in interviews. At one site, an interviewee helped us to set up a focus group with 9 practice nurses as part of an immunisation training day. We endeavoured to repeat this at the other sites but were unsuccessful. Most of the interviews were conducted individually, apart from three peer interviews with 2–3 people from LA public health teams and the focus group with providers. Nine interviews were conducted over the telephone at the request of participants, others took place in private spaces within places of work.

### Data analysis

Interviews were audio recorded and transcribed verbatim, with only 4 participants opting for notes to be taken during the interview. These notes, written accounts of observed events and the interview transcripts were imported into a qualitative data analysis programme (NVivo10). The approach to analysing this data was primarily inductive, which meant that we sought to be attuned to emerging themes rather than just pursuing the pre-defined interview topics. Three researchers met regularly to discuss emerging themes, resolve discrepancies in data analysis, confirm definitions of higher level themes and sub-themes, and produce a consistent framework that was systematically applied to the whole data set [34]. Our preliminary analysis and findings were also presented to the wider study team for verification. The study received ethical approval from the LSHTM Ethics committee (Ref: 8661) and obtained NHS Assurance support (IRAS project ID 164911) for the local sites.

## Results

Our data are presented under three overarching themes: 1) transition to the new health system; 2) applying the new arrangements for immunisation; and 3) regrouping and making the new arrangements work. These themes narrate participants’ experiences of the transition of the immunisation programme into a new health system structure, document how they learned to work within the new management and delivery arrangements in this structure and identify the mechanisms they used to make these arrangements work.

### Transition to the new health system

This theme documents participants’ initial experiences of the transition process and how they sought to understand, adapt and make sense of organisational and individual responsibilities for delivering the immunisation programme within a new health structure.

### Fragmentation in the delivery of the immunisation programme

Rhetorical devices like *"in the old world"*, *"in the PCT world"*, and now *"in the new world"* were used by many interviewees to describe the transition to a different health system, and convey the extent of change that had occurred in the organisation of the immunisation programme. The reallocation of immunisation functions across new or reformed organisations was viewed as having fragmented the delivery of the immunisation programme.

*“Since April last year (2013), this system of immunisations is fractured; it really is fractured. So, you’ve got Public Health England, and the Department of Health and the JCVI creating the strategy or policy; you’ve got NHS England commissioners … trying to implement, and then at the side of that you’ve got local authority colleagues holding us to account for assurance purposes … Three organisations are trying to inspire general practice or primary care, or providers, to jab more. It’s a complex mesh, so it’s trying to hold that mesh together, at the moment.” (NHS England, 59)*

Interviewees reported that immunisation, as a public health programme, did not slot neatly into the new health structure. To quote a national stakeholder, (19) it was “*the bit that didn’t fit.”* According to participants, it took a significant amount of deliberation to decide a way forward. These decisions included retaining responsibility for immunisation within the NHS, even though the 2012 HSCA had delegated the management of public health programmes to local government, and embedded PHE-led screening and immunisation teams within local NHS England commissioning bodies. These teams in turn had to develop effective working relationships with partners in LA Public Health Teams, CCGs, and PHE Health Protection Teams in order to make sense of the new delivery arrangements for immunisation. This dispersal of responsibilities across multiple organisations raised questions about: *“who’s got that overarching leadership and accountability.” (LA Public Health Team, 44)*

Whilst changes to the provider landscape were viewed as having created opportunities for testing new ways of delivering certain programmes, they also resulted in some schools having to host different immunisation providers for different school age vaccination programmes (e.g. school leaver boosters, cervical cancer, flu). One commissioning manager (SIT, 48) described changes to the provider landscape as *“a second level of fragmentation*”, and highlighted the risks fragmentation posed to effective communication with parents and schools, and between partners in the management of contracts and data.

According to those involved in managing and assuring the quality of the immunisation programme, this kind of complexity required them to *“work very hard to pull it [the system] back together” (LA Public Health Team, 28)*, and streamline processes within and across organisations in order to *“bring them together somehow.” (SIT, 48).*

### Redeployment and shifts in working practice

The implementation of the health reforms resulted in a significant movement of human resources in terms of teams, organisations or individuals. For some this involved a loss of independence, a change in contract and working culture, and a move from a technical to a more political role. For example, Directors of Public Health (DPH) were removed from PCTs and tasked with establishing public health teams within the much more political arena of local authorities. This required them to negotiate public health priorities and funding decisions with elected council members whose primary business is not health. In terms of contracts, staff who transferred from the Health Protection Agency to PHE became civil servants (albeit in most cases on NHS terms and conditions), which was reported to have had wide ranging repercussions, including increased scrutiny of publications and reduced freedom to question national government policy decisions. Reflecting on this cultural shift, one participant felt that PHE had become an *“upward facing, not outward facing”* organisation with different priorities:

*“…having to answer Parliamentary questions, and briefing Ministers, and it’s…because we’re civil servants, that’s seen as a bigger priority than supporting the frontline, which is a huge cultural shift that I don’t feel comfortable with, because I see my job as supporting the frontline, because I want children to be vaccinated.” (National interviewee, 4)*

Interviewees’ experience of staff redeployment was shaped by where they moved to, whether they moved with a team or alone, and how much their role changed. Moving from DH into PHE as part of the immunisation implementation team was described as disruptive but manageable. Moving to NHS England was experienced as more challenging since this new organisation had to rapidly assume responsibility for commissioning the delivery of Section 7a programmes. Moving from an active role in immunisation activities in PCTs to more indirect support in local authorities required individuals to acquire new skills in assurance and relinquish valued hands-on duties, such as implementing projects aimed at increasing the uptake of immunisation in vulnerable populations. Moving from PCT immunisation teams to SITs was described as less challenging since the work was similar, albeit on a wider scale, with new commissioning responsibilities, and less *"hands on"* capacity for interacting with immunisation providers.

When describing the process of redeployment at local level, interviewees talked about being *“slotted and matched”*, or “*shifted and lifted”.* For some, this involved competing for positions that suited their skills, for others a straightforward transfer occurred, and a few people ended up being put at risk or made redundant since an equivalent role could not be identified. For SIT leads, key challenges were finding staff with skills and experience in immunisation, screening and commissioning, and *“developing a team, that is embedded within NHS England employed by Public Health England, and that ultimately don’t feel like they belong in either*” *(SIT, 65)* A significant consequence of the redeployment was the removal of budgets and decision-making from local players to regional ones and a loss of local knowledge (the historical memory gained from working in an area for a long time and the relationships built over time between providers and service managers), insights into underperforming areas and practices, and the understanding of contextual factors that affected the uptake of immunisations. In one LA, a DPH sought to mitigate this loss by assigning a former PCT immunisation coordinator the responsibility for *"keeping an eye on what was going on with immunisation and keeping a steady ship" (Provider & LA Public Health Team, 69)*.

### Adapting to the new infrastructure

Adapting to the new modus operandi for immunisation required people to revise previous patterns of working, adopt new roles and responsibilities, acquire new skills and make new connections. Many interviewees found it difficult to establish new working rhythms and commented on how long it had taken for the system to settle.

*“We’ve been here nearly two years and it just about feels we’re beginning to manage it appropriately.” (SIT, 23)*

A couple of years in, many interviewees were still grieving for their old jobs, particularly if their redeployment had resulted in a loss of autonomy, or left them less able to improve practice or influence policy.

*“We had far more autonomy and far more responsibility and it was great. It was a really satisfying job actually and it was great to feel that you’d managed to get those figures, those rates up in that specific area.” (SIT, 41)*

One participant (LA Public Health Team) also suggested that the effort expended in adapting to the new system obscured opportunities for improved practice, and made people more reticent about ongoing structural changes (e.g. CCG ‘co-commissioning’ of primary care). Mechanisms for coping with the change included establishing contact with past immunisation colleagues who had moved to different organisations and building informal relations alongside official channels in order to establish the partnerships, which were perceived by participants as core to the management of the new system. From a provider’s perspective, it meant turning to the people who used to provide advice even if this was no longer in their remit.

### Applying the new arrangements for immunisation

This theme documents participants’ experiences of learning to implement the new arrangements for the immunisation programme in a more complex health infrastructure.

### Tripartite working at national level

One of the most significant changes at national level was the introduction of tripartite working. Immunisation was no longer solely led by DH, instead accountabilities were shared with NHS England and PHE. This required national leaders to develop a completely different way of working: whereas previously policies had been agreed and executed by one organisation in a *‘command and control style’*, they were now reviewed by partners who provided detailed input on implications for implementation and commissioning. Although the new governance arrangements made rapid responses to public health contingencies more challenging, annual revisions of Section 7a agreements were viewed by some as having helped national partners make sense of tripartite immunisation planning and cross-organisational collaboration.

“*We’ve got strong governance arrangements in place to support the delivery of the 7a agreement that locks everybody into a way of working that ensures we work collaboratively together in a strategic way.…The Section 7a agreement forces you to have a proper strategic conversation with the NHS… whereas that didn’t really happen.” (National interviewee, 8)*

Despite the emphasis placed on joint responsibility, questions arose about how to manage mutual accountabilities. Diverse opinions were tendered about which organisation wielded greatest influence, with some attributing greater command to DH, as the delegating authority, and others to NHS England, because of its responsibility for the assigned budget. The process of clearing and checking each other’s contributions to official correspondence was mentioned as an example of difficulties encountered in balancing power and exercising trust in tripartite relationships.

“*Under tripartite working all three organisations have equal rights to change the letter…so that would delay things…and the kind of “I must be the last to sign this off” syndrome is very much existing in all three organisations.” (National interviewee, 15).*

### Applying the local operating model for immunisation

The application of operational guidance for the immunisation programme at local level was not straightforward, according to a wide range of interviewees. The dispersal of duties and the creation of new teams and roles resulted in a lack of clarity and varying interpretations as to who was responsible for what, and how the system should be implemented collaboratively.

“*There was a lack of* clarity *about what do these new roles actually mean … Okay, we can say, well, ours is the assurance role and the area team commissions, but actually in terms of divvying up the tasks, what does that mean, who does what, how does it come together and make a whole?” (LA Public health team, 27)*

“… *there’s an operating framework, there’s job descriptions and, as I said, I think it’s absolutely clear within that what we’re supposed to be doing, but people are not working in those ways and I think there’s different interpretations.” (SIT, 65)*

The management of vaccine preventable disease outbreaks was cited as an example of where there was a lack of clarity about responsibilities; i.e. who should lead the response, who should be mobilised to immunise or provide chemoprophylaxis, and who should cover the costs. Similarly, the existence of different organisational reporting procedures was viewed as having complicated the management of incidents such as errors in the administration of vaccines or failures in cold chain storage.

LA Public Health Teams found discharging assurance responsibilities challenging due to limited access to data and a lack of operational involvement in the immunisation programme, including an inability to take part in outreach to under-vaccinated local populations. They also had to ensure that any immunisation support they provided to CCGs, as part of their core public health function/intelligence work, did not overlap with SITs work.

SITs, which had been envisioned as a public health resource within NHS England ATs, reported that they were less able to apply their clinical expertise and were more focussed on commissioning and logistics.

*“I think they saw us as just extensions of their commissioning team, and I felt that my professional role was being dumbed down from band 7 clinical specialist to band 4 admin assistant, because the, I think, the feeling in NHS England was as long as there’s a contract, everything’s good.” (SIT, 18)*

SITs increased ‘footprint’ (the term used by participants to denote their geographical areas of responsibility), and difficulties in recruiting and retaining appropriately qualified staff had limited their ability to support immunisation providers. Consequently, they were not confident about understanding provider performance, and thus less able to monitor and reinforce good practice.

*“…we are trying to solve issues that we don’t fully understand because we don’t actually have the resource to go out there and do the investigative work that is required. So we are, in a way, working blindly.” (SIT, 40)*

On the other hand, SITs larger ‘footprint’ was thought to have supported a more equitable distribution of resources and strengthened commissioning processes by introducing a more consistent approach across larger geographical areas. Several SIT members also stated that the broader *“helicopter”* or *“pan area”* view enabled them to identify and share good practice across localities facing similar problems.

Although their role had been less affected by the changes, immunisation providers generally found it difficult to access advice, support and training in the new system, and many were unclear about the differences between SITs and LA PH teams. Providers of school-based immunisation programmes were more affected by the changes since they now had to tender for contracts. Decisions regarding tenders were usually made by LAs but NHS England commissioners could also be consulted, and this led some community providers to feel less able to discuss operational problems openly with SITs.

Reflecting on his experience of working in the new system, an interviewee from a LA Public Health Team (66) described how *“everyone feels very insular in all sorts of ways”*; each organisation attended to its own responsibilities, which could be positive, but in the absence of effective collaborative processes, this could also increase the potential for *“territorial silo issues”* and *“friction”*.

### Regrouping and making the new arrangements work

This theme describes what participants did in order to be able to deliver the immunisation programme in the new more complex health care system and take any opportunities presented by the new system.

### Working in partnership: “To join up different bits of the system”

Interviewees underscored the need to build effective collaborative processes and strong relationships to make the national framework and local operating model work tolerably well. Establishing and maintaining partnership working reportedly required significant time, effort and creativity but it also increased programme accountability and created opportunities for sharing good practice and troubleshooting. Some mechanisms for partnership working were set up as part of the implementation of the HSCA in April 2013, others were developed more iteratively. Examples of the former were the National Immunisation Programme Board (IPB) and LA Health Protection Forums. These governance structures proved useful for supporting strategic collaboration, but often needed to be complemented by more operational committees, for example, a newly formed national implementation group.

*“The Health Protection Forum wants to make its priorities things that it can do together, so the whole point is that different people are responsible for different bits of the system now, and there is some fragmentation. But obviously there are lots of areas that we all need to work together on, so that forum is a way strategically of joining up some of those dots.” (LA Public Health Team, 28)*

Whilst Health Protection Forums were recognised by local participants as a core mechanism for partnership working, they were not the only means used to foster multi-agency collaboration in improving local immunisation outcomes. At Site 1 (see Table 2), regular strategic meetings between senior SIT members and LA DPHs were organised, and four pre-existing immunisation committees re-appointed. SIT and LA public health leads felt the latter had provided opportunities to commence constructive conversations, and a community provider valued the transparency and joint problem solving they facilitated. However, difficulties were reported in achieving CCG and general practice committee representation.

*“I think we’re struggling to make the collaboration work, because we’ve been finding our feet, the local authorities have been finding their feet and the CCGs are also doing it, so I do not think that we’ve got it right. We’ve made progress, we have conversations. I think now that we begin to understand a bit more about where we’re going, we can have better conversations.” (SIT, 23)*

At site 2, the SIT established an immunisation board with senior representation from NHS England, CCGs, PHE health protection teams, academia, pharmacy, LA Public Health Teams and NHS Trusts. A local partnership component was added in 2015, with each LA area asked to agree an action plan for improving immunisation coverage. Initial experience indicated that this worked best in areas where there were existing local immunisation groups (carried over from pre 2013), or where immunisation was a standing item at Health Protection Forums. Other local areas were more resistant about accepting responsibility for leading plans and owning actions, and some expected a separate budget to underpin this work.

*“But it’s, from the perception I get from some local authorities, it’s like it’s your responsibility NHS England, what are you doing about it? But we can’t be out there on the ground because that’s not our role. Our role is as commissioners, we’re contracting, we’re providing service providers to do it. We’re working in partnership with you. So it’s all our roles to ensure this happens.” (SIT, 73)*

At Site 3, Health Protection Forums were cited as the main means of promoting partnership working across the AT. In addition, SIT members attended flu vaccine provider meetings run by CCGs and were asked to support a pre-existing district immunisation committee run by a paediatrician and a practice nurse. A new committee involving different CCGs and LAs was also planned to help reverse historically low immunisation uptake rates in one area.

National and local level interviewees agreed that the success of the immunisation programme hinged on developing strong working relationships with key individuals based in different organisations. This was challenging for SITs which covered a large number of LAs and CCGs, and difficult for national partners who had limited opportunities to meet in person and who communicated mainly by email or phone. The importance of face to face workshops as a means of nurturing trust “… *building the sort of confidence and individual relationships up which I think is very important to any of this’ (National interviewee, 12)* was highlighted*,* and partnership skills training identified as an important workforce development programme.

“*I think for a future workforce it is really about bearing in mind that partnership working is part of someone’s job description…being able to have that knowledge of tapping into those different structures and things. I think that is a core skill… to promote the uptake of immunisation.” (LA PHT, 27)*

### Building on opportunities and addressing gaps

Professionally led SITs embedded within NHS England ATs were considered to be an important resource and potential strength of the new system. National leaders have supported them by running fortnightly teleconferences and six monthly meetings for team leads. SITs dual accountability to PHE and NHS England was however also viewed as having contributed to difficulties in defining their role, and achieving the right balance between commissioning and supporting providers. This lack of definition was maintained to have resulted in a huge variation in the way SITs operate. Many SITs had also been functioning below capacity due to staff attrition and problems in attracting professionals with the right skill sets to civil service posts. This lack of capacity and SITs increased footprint has had a knock-on effect on SITs ability to respond to local needs and performance manage immunisation providers. NHS England has sought to address the latter by providing SITs with real time immunisation uptake statistics via a data management system, and data sharing agreements have also been developed to enable LA Public health teams fulfil their assurance responsibilities. The following mitigating strategies were also observed at local level: 1) at Site 3, a CCG had allocated funding to immunisation as a priority area and established an influenza immunisation service for nursing home residents; 2) at all sites, the LA Public Health Team linked SITs with schools and community based children’s centres (tailored services for families with children ≤5), and 3) at Site 3, the SIT had enlisted the support of CCG quality improvement staff to enhance provider performance and a CCG had independently appointed an infection control nurse to assess the quality and uptake of immunisation services in GP surgeries. These local strategies, though well intentioned, tended to be informal and in the case of the last, was short lived since funding constraints meant that the role was not sustained.

The ad hoc manner in which problems tended to be resolved was even more apparent in relation to the provision of training for immunisation providers. The local operating model was not clear about the role SITs should play in helping health care professionals and their employers ensure that they had been trained in accordance with the mandatory requirements.

*“…what does facilitate mean? It doesn’t say who’s actually responsible. So yes, the SIT could be responsible for facilitating training, but that doesn’t necessarily mean to say they’ve got to do it.” (National interviewee, 18)*

Sites adopted different approaches to fill this gap. At Site 1, CCGs and the SIT lobbied local universities to provide essential skills courses for practice nurses that would cover immunisation, and CCG practice nurse leads either secured internal funding, or bid for Local Education Training Board funds to be able to conduct immunisation training at protected learning events. At Site 2, the SIT commissioned health care academics to provide introductory courses and the health protection team were starting to provide updates, while at Site 3, practice nurses had set up monthly training sessions which were supported by their CCG and a management company*.* There was recognition that, whilst these initiatives helped fill critical gaps, it might not be possible to replicate them elsewhere.

“…*there is huge inconsistency about [training] provision, including no provision, and there is a lack of clarity and a lack of understanding about who should be providing it, who should be commissioning it and who should be funding it.” (National interviewee, 18)*

Concerns about inconsistencies in the delivery of the immunisation programme were raised by many interviewees. In addition to clarifying roles and functions and strengthening governance processes, a few interviewees suggested that a redistribution of roles might be necessary. For instance, a national interviewee argued for a strengthened role for CCGs to use their position as local peer leaders to oversee and encourage improved uptake. Aversion to further change however dampened local implementers’ responses to these types of suggestions.

## Discussion

The purpose of this research was to analyse in depth how the national immunisation programme had adapted to changes in the institutional environment and identify what had facilitated or hindered the delivery of immunisation services in this context. Our findings indicate that the April 2013 health system reorganisation in England resulted in significant fragmentation in the way that the immunisation programme was managed and delivered. This appeared to be an unintentional by-product of the 2012 HSCA [12], and it required national and local partners to work hard to reintegrate the delivery of the immunisation programme. Adapting to the new structural arrangements was handicapped by the redeployment of experienced immunisation professionals across new and revised organisations. This movement resulted in a loss of local and historical knowledge especially when staff moved to different areas. WHO describes a similar problem in relation to health system reforms in general and makes the following recommendation about maintaining institutional memory: ‘*While developing new structures and systems, retain the lessons learned from a particular programme; consider past experience in the context of future possibilities (p.22)’*[5]. In the current study, it was easier to maintain institutional memory at national rather than local level mainly because many pre-reform leaders maintained similar strategic positions post April 2013, albeit within new or revised organisations. At local level, former colleagues had to reconnect in new ways in various partnership forums.

It took more than two years for the mechanisms for the delivery of the immunisation programme to settle and many interviewees grieved for the former system as they adapted to new ways of working. This period of protracted adjustment and associated low morale is consistent with other evidence, for example, about the effects of hospital mergers, and of the relationship between organisational change and health professionals’ well-being [23, 24, 35]. Furthermore, immunisation is a population-based public health programme that needs to achieve high coverage rates in order to prevent disease. Hence, it requires strategic coordination and consistent, integrated delivery mechanisms; characteristics that are less critical in more demand-led health services that cater for individual patients’ medical concerns. The dispersal of immunisation functions across different organisations, and the lack of clarity and variable interpretations about roles and responsibilities compounded these difficulties. Previous research has shown how this type of organisational confusion can undermine health system reform and lead to further restructuring [25]. Our interviewees were concerned about the possibility of further restructuring and did their best to hold the system together and work around emerging gaps in the system. As a result, these gaps (e.g. provision of training) were not addressed in a coordinated manner. Instead, there was a tendency to rely on the goodwill of staff to develop their own local solutions to weaknesses in the programme. Furthermore, although the new arrangements were reported to have resulted in a greater consistency and efficiency in commissioning across wider geographical areas, they also reduced managers’ capacity to interact directly with immunisation providers. This distancing is one of the most striking differences between the old and the new immunisation system. The loss of core roles (e.g. in particular, the PCT immunisation coordinator) removed focal reference points for providers, and rendered performance evaluation and support more challenging. According to the local operating model, screening and immunisation teams were to become providers’ new reference points but these teams prioritised commissioning and contractual outcomes over clinical leadership.

Our results are consistent with literature that highlights the disadvantages of large-scale organisational change versus incremental change which supports continuous improvement [20, 23]. The sudden fragmentation of immunisation activities between many organisations suggests that immunisation fell through the cracks in the development of the changes, and was not adequately considered between publication of the HSCA and its implementation. The key focus of the 2013 NHS reform was re-organising commissioning of clinical care and delegating public health to local authorities. In effect, the complexity of the resulting organisational landscape and the lack of alignment between the pre- and post-reform immunisation delivery arrangements meant that systemic challenges could only be addressed by rebuilding effective partnerships informally at local level.

### Strengths and weaknesses

In studies analysing the effects of organisational change on service delivery, it is important to account for individual and contextual factors that can affect the implementation of change. We sought to do this by examining the national framing of the service reorganisation and its application across different levels of the immunisation system over a period of a year. Collecting data over a period of a year allowed us to document how individuals and organisations adapted over time, and how different sites responded to particular challenges. Our sample of interviewees included a wide range of actors at macro and meso levels of the health system, who were differentially affected by the re-organisation. In this paper, we focussed on national leaders and local managers because they experienced the most change and were responsible for maintaining strategic oversight and ensuring immunisation services continued to be commissioned and provided locally.

This is a qualitative study hence our findings are not statistically generalisable but they are indicative of the processes produced by a system wide reorganisation. The study was not designed to look at whether the re-organisation had had a direct effect on immunisation coverage. This would have required a different design. To address these limitations and to quantify the phenomena captured qualitatively, the second phase of the research programme includes a nationally representative questionnaire survey of professionals involved in delivering the national immunisation programme in England which took place in July and August 2016, and will be reported in due course.

### Implications of our findings

Our findings have direct implications for the management of the English immunisation programme and accentuate the need for international policy makers to be more alert to the ways in which health system reorganisation can affect constituent public health programmes.

### National immunisation programme management in England

The fact that vaccination coverage in England overall remained relatively stable in the years following the April 2013 health system reorganisation is credit to the diligence of programme planners, commissioners and providers [36]. However, to protect and enhance delivery, attention needs to be paid to developing system-wide strategies for addressing weaknesses; notably, access to regular training and proactive performance evaluation which enables providers to identify and share good practice. Without advocating more structural changes, we think that there is a need for improved utilisation of different partner organisations’ strengths. LA Public Health Teams and CCGs that are responsible for smaller geographic areas than SITs could do more to strengthen outreach to under-vaccinated communities and review the performance of their constituent practices, respectively. We appreciate that this is not straightforward given differing views about the transfer of the public health function from the NHS to local authorities [37, 38], and the lack of organisational uniformity across CCGs [39]. Finally, given the relevance of partnership working, staff need be given the opportunity to acquire relevant skills and reflect on past experience, in order to build and nurture collaborations needed to ensure the continued success of the national immunisation programme.

### Wider implications

Policy makers, politicians and their advisors need to heed the warnings about the futility of regular large-scale health system reforms [10, 20]. The key message is for them not to rush in and disrupt something that is working well, instead, to target specific programmes that could benefit from incremental improvements. Taking this approach will help retain institutional memory and local knowledge that is critical to the functioning of effective and efficient public health programmes. With reference to partnership working, more evidence is needed about the effectiveness of different forms of macro- and meso-level collaboration in terms of achieving health care outcomes (e.g. increasing vaccine uptake in under-served populations) [40].

## Conclusions

The April 2013 health system reorganisation in England resulted in significant fragmentation in the way that the immunisation programme was commissioned and delivered. While some of this was a temporary by-product of organisational change, there were more persistent challenges intrinsic to the complex architecture and governance of the health system envisioned in the 2012 HSCA and implemented from April 2013. Partnership working helped immunisation leaders and implementers reconnect during the transition phase and now the challenge is to assess how inter-agency collaboration can be strengthened in order to ensure that programme performance is maintained.

## Additional files

Additional file 1: National level key informants interview topic guide. This file provides the range of topics and questions that were covered in interviews with national participants. (PDF 219 kb) Additional file 2: Immunisation service managers and partners interview topic guid. This file provides the range of topics and questions that were covered in interviews with people involved in commissioning, managing or assuring the immunisation programme at local level. (PDF 332 kb) Additional file 3: Immunisation service providers interview topic guid. This file provides the range of topics and questions that were covered in interviews with health professionals involved in delivering immunisations in primary care and as part of community care organisations. (PDF 315 kb)

## Abbreviations

AT: Area team
CCGs: Clinical commissioning groups
DH: Department of health
HPA: Health protection agency
HSCA: Health and social care act
LA: Local authority
LA PHT: Local authority public health teams
NHS E: NHS England
PCTs: Primary care trusts
PHE: Public health England
SIT: Screening and immunisation teams
