# Supplementary material for: “It’s a complex mesh”- how large-scale health system reorganisation affected the delivery of the immunisation programme in England: a qualitative study
Source: BMC Health Serv Res. 2016 Sep 15;16:489. doi: 10.1186/s12913-016-1711-0 (PMC5025625; doi:10.1186/s12913-016-1711-0)
Supplement: Additional file 1: — National level key informants interview topic guide. This file provides the range of topics and questions that were covered in interviews with national participants. (PDF 219 kb) [file 12913_2016_1711_MOESM1_ESM.pdf]

Interview Topic Guide Notes: National Level Key Informants

Time: 45-60 minutes

Questions in these interviews will depend on the nature of the key informant and their position and therefore will be developed prior to the interview with the aim of gaining:

1) An in-depth understanding of the roles and responsibilities of senior leaders and their respective organisations with regards to the current immunisation programme;
2) A good understanding of how immunisation programme functions, is managed and overseen from a policy perspective;
3) A good understanding about how immunisation related policies are developed, authorised and evaluated;
4) Insights into the involvement of key informants and their respective organisations in planning and executing the April 2013 changes to the immunisation programme;
5) An overview of what changed, why and how;
6) Insights into key informants' opinions and experiences of these changes and their views on how they were implemented;
7) Insights into key informants' opinions and experiences about what worked well, what did not work so well, how this was resolved, and whether any policy adaptations had to be made due to operability problems**;**
8) Insights into what key informants think about how are the new arrangements are working (are the right structures are in place and working? If not, where are the problems? Are there challenges? Are the challenges (if any) being addressed, in what way? Are there any opportunities that can be optimised? Are they being taken up?);
9) An understanding of how partner organisations collaborate in delivering and managing the immunisation programme;
10) Insights into differences in structure and working culture between different partner organisations and how this influences collaboration;
11) An understanding of key informants' responsibilities and views about the performance evaluation of the immunisation programme;
12) Insights into other topics which arise in discussion and are considered as important by interviewees.

At the end of the interview the interviewer will ask the key informant if they have any recommendations in terms of other people we should speak to, if they can share any relevant documents with us, and if there are any relevant meetings or immunisation related activities we could observe.

Finally, the interviewer will thank the key informant for their time.
