# Supplementary material for: “It’s a complex mesh”- how large-scale health system reorganisation affected the delivery of the immunisation programme in England: a qualitative study
Source: BMC Health Serv Res. 2016 Sep 15;16:489. doi: 10.1186/s12913-016-1711-0 (PMC5025625; doi:10.1186/s12913-016-1711-0)
Supplement: Additional file 2: — Immunisation service managers and partners interview topic guid. This file provides the range of topics and questions that were covered in interviews with people involved in commissioning, managing or assuring the immunisation programme at local level. (PDF 332 kb) [file 12913_2016_1711_MOESM2_ESM.pdf]

Interview Topic Guide: Immunisation Service Managers and Partners

(NHS England, Local Authority Public Health Team members, PHE England Staff, CCG members)

Time 45-60 minutes

Current practice: What is happening, and how?

<u>About you and your organisation's work</u>

Tell me about your current role and how it relates to immunisation. Who do you work with, who do you manage and who are you accountable to?

Please describe your day to day immunisation related activities (e.g. planning and implementation, commissioning, specialist advice, logistical leadership, monitoring and scrutiny, data management)

What are your, and your organisations main responsibilities in terms of the immunisation programme? Which agencies (both internal and external) do you work to carry out immunisation activities?

- Name of organisations/services and examples of how you work together
- Are there formal structures through which you work together? *(for example, if through a committee, are there any TOR, when was it set up, frequency of meeting)*

How is the local area you cover or are based in performing against local and national immunisation standards? (Has this always been the case?)

What have been the main successes and the main challenges over the past year?

What has changed?

How have the changes to the organisation of the NHS, which came into operation in April 2013, affected you and your organisation's role and involvement in the immunisation programme?

What has changed for you? What has changed for your organisation?

In terms of new responsibilities and accountabilities

In terms of working with other agencies

In terms of leadership and oversight

In terms of commissioning

In terms of managing and reporting immunisation data

In terms of the evaluation of vaccination service performance

In terms of accessing and providing training and support

In terms of the management of outbreaks and clinical incidents related to immunisation

In terms of responding to public concerns of complaints

How was/is change being managed?

How were these changes introduced and managed? What worked well, what was difficult? What could have been done better?

Tell me about your experience of these changes. How have you personally adapted? How did your organisation adapt, and what have the consequences been?

What support was available to manage the process of change?

What is your understanding on the local arrangements, structures and responsibilities for immunisation following these changes? How are these new arrangements working?

What structures are in place to achieve oversight, monitoring and coordination of services (e.g. a local strategy and/or implementation committee)? Are the responsibilities of those involved clearly defined?

What do you think about these structures? How are they working? What is going well? What is not going well, where are the problems and how could these be resolved?

- Are there any challenges (e.g. IT systems, collaboration with GPs/stakeholders, implementation of new vaccines)? Are the challenges (if any) being addressed, in what way?
- Are there any opportunities that can be optimised (e.g. role of LA in reaching unregistered populations, commissioning)? Are they being taken up?

Reflections on change

Given your experience, what are your views on the changes to the immunisation programme that came into effect in April 2013?

Any other comments

Do you have any other comments?
Is there anyone else you would recommend I should to speak to?
Are there any relevant meetings of immunisation related activities I could observe?

Thank you for your time.
