# Supplementary material for: “It’s a complex mesh”- how large-scale health system reorganisation affected the delivery of the immunisation programme in England: a qualitative study
Source: BMC Health Serv Res. 2016 Sep 15;16:489. doi: 10.1186/s12913-016-1711-0 (PMC5025625; doi:10.1186/s12913-016-1711-0)
Supplement: Additional file 3: — Immunisation service providers interview topic guid. This file provides the range of topics and questions that were covered in interviews with health professionals involved in delivering immunisations in primary care and as part of community care organisations. (PDF 315 kb) [file 12913_2016_1711_MOESM3_ESM.pdf]

Interview Topic Guide: Immunisation Service Providers

(Time: 30-45 minutes)

Current practice: What is happening, and how?

<u>About you and your role</u>

Tell me about your role in immunisation at your practice/place of work. What are your main responsibilities, who do you work with and who are you accountable to?

<u>Immunisation service delivery</u>

What vaccination services are offered at your practice/place of work?

How are these services organised and delivered at your practice/place of work?

How do you review and assess the performance of your immunisation services? Do you set immunisation targets (if yes, how and what are these)?

How do you record and store immunisation data, what electronic data systems do you use and who do transfer this data to?

What have been the main successes and the main challenges over the past year?

What has changed?

How have the changes to the organisation of the NHS, which came into operation in April 2013, affected you and your organisation's role and involvement in the immunisation programme?

What has changed for you? In terms of your role.

What has changed for your practice/place of work?

In the way you plan vaccination services (e.g. accessing vaccines, scheduling)

In the way you run vaccination services (e.g. staff involved)

In the terms of the organisations/professionals you work with (e.g. immunisation leads, health visitors)

In the reporting and transfer of immunisation data (e.g. new systems, managing data)

In the way that vaccination service performance is evaluated

In the way that you access training and support

In the way you deal with clinical incidents related to immunisation

In the way you report and are involved in the management of outbreaks

In terms of responding to public concerns of complaints

How was/is change being managed?

How were these changes introduced and managed? What worked well, what was difficult? What could have be done better?

Tell me about your experience of these changes. How have you personally adapted? How has the practice adapted, and what have the consequences been?

What support was available to manage the process of change?

How are the new arrangements working now? What is working well, what is challenging and how do you try to resolve these challenges?

What do you know about and what do you think about the way that the immunisation programme is managed at Area Team, Local Authority level? What degree of interaction do you have with these organisations and others like Public Health England?

Reflections on change

Given your experience, what are your views on the changes to the immunisation programme that came into effect in April 2013?

Any other comments

Do you have any other comments?
Is there anyone else you would recommend I should speak to?
Are there any relevant meetings of immunisation related activities I could observe?

Thank you for your time.
